# The Use of Hydroxyapatite Polymer with Curdlan in the Treatment of Bone Defects Associated with Ectopic Tooth Extraction in Dogs—A Case Series

**DOI:** 10.3390/life14070879

**Published:** 2024-07-15

**Authors:** Anna Misztal-Kunecka, Przemysław Prządka, Stanisław Dzimira

**Affiliations:** 1 Veterinary Clinic DentalVet Anna Misztal-Kunecka, 71-795 Szczecin, Poland; 2 Department and Clinic of Surgery, Faculty of Veterinary Medicine, Wroclaw University of Environmental and Life Sciences, 50-375 Wroclaw, Poland; przemyslaw.przadka@upwr.edu.pl; 3 Department of Pathology, Division of Pathomorphology and Veterinary Forensics, Faculty of Veterinary Medicine, Wroclaw University of Environmental and Life Sciences, 50-375 Wroclaw, Poland

**Keywords:** ectopic tooth, hydroxyapatite polymer, curdlan, dental cyst

## Abstract

Ectopic teeth are an eruption disorder in which teeth are located in anatomical structures where, physiologically, they should not occur. An ectopic tooth is a very rare phenomenon, affecting approximately 0.5% of the canine population, and few descriptions of the treatment of such teeth in dogs can be found in the available literature. This article describes the diagnostic and therapeutic handling of cavities following extraction of ectopic teeth in nine dogs. The cases are subdivided into uncomplicated (when the ectopic tooth was encapsulated in the surrounding connective tissue, without lysis of the bone around the tooth) and complicated (in which, in addition to the presence of the ectopic tooth, a dentigerous cyst had formed). Four cases of complicated ectopic teeth are described in more detail. In this study, special attention was paid not only to the technique of tooth extraction itself but also to the method of securing and healing large bone defects after the extraction using hydroxyapatite curdlan polymer. Owing to the plastic properties of the bone substitute preparation, it was possible to implant the material without enlarging the bone defect created during the tooth extraction. Control radiographs showed features of bone regeneration, and clinical examination at both the early and late stages revealed no postoperative complications.

## 1. Introduction

Tooth development is the result of a complex, multi-stage interaction between the oral epithelium and the underlying mesenchymal tissue. Abnormal tissue interactions during development can result in ectopic tooth eruption and development. Ectopy is a disorder of tooth eruption that involves the location of a tooth or tooth bud within anatomical structures where teeth should not occur [1]. An ectopic tooth can be observed on the palate, the beak process of the mandible, in the eye socket, in the septum, or in the nasal cavity. Etiological factors include genetic conditions, abnormal eruption of permanent teeth, crowding of permanent teeth, and an overly compact jawbone structure [2]. According to Renger et al., up to 58.7% of ectopic teeth fail to erupt, suggesting that anatomical or histological obstacles related to bone density are one of the causes of this condition [3]. They most often occur unilaterally and more often affect single-rooted teeth than multi-rooted teeth, which, according to Bellei et al., may play a role in stopping the eruption process [4].

The diagnosis of an ectopic tooth is based on the dog’s history, clinical examination, and radiological findings. An X-ray examination is sufficient to confirm the diagnosis and to confirm the location of the ectopic tooth [4].

**Figure 1:**
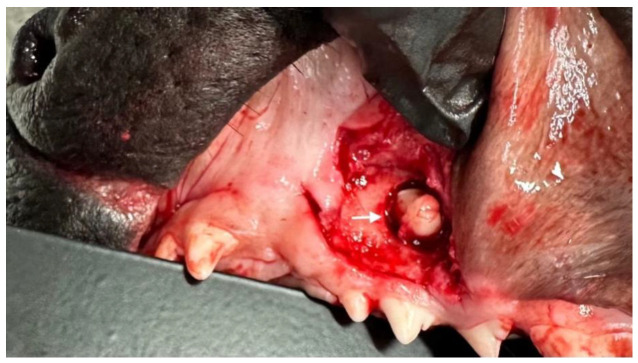
Surgical access for extraction of an ectopic tooth. The ectopic tooth is marked with an arrow.

The extraction of an abnormally located tooth poses a major challenge to surgeons, not only because of the location but also because of the resulting large bone defect after extraction. Two techniques for the extraction of ectopic teeth in the jaw are described in the literature. The first is the surgical method (CLP or the Caldwell–Luc Procedure) and the second is the endoscopic method, which is currently reserved for human medicine [5]. The removal of ectopic teeth using the CLP technique involves creating access to the sinus in the oral vestibule from the frenulum of the upper lip to the region of the premolar/molar teeth (Figure 1). Once the access is made, the altered mucosa and cystic follicle are removed. The endoscopic technique was designed to minimize tissue loss, especially bone tissue during extraction, but has many limitations in its use in veterinary medicine. The planning of surgical extraction should also take into account the long-term consequences of the procedure, which may be a weakening of the jawbone resulting from the removal of such a large structure and the need to cure the capsule in which the ectopic tooth is located. In order to fill the cavity, the use of a bone graft or, more conveniently for the surgeon, the use of a ready-to-use bone graft material, which also has the advantage of not increasing tissue trauma for the dogs, seems to be necessary. The available hydroxyapatite-based materials are quite commonly used for this purpose. Hydroxyapatite (Ca_10_(PO_4_)_6_(OH)_2_) is the main mineral component of bone and teeth, providing a scaffold for newly formed bone tissue. HAp-based preparations are widely used in human regenerative medicine and are also increasingly applied in veterinary medicine [6,7,8]. The use of pure hydroxyapatite is not a good solution as the increased amount of hydroxyapatite in the implant material reduces the rate of material degradation, as mentioned by Jung, B.T. et al. Hence, according to these authors, it is necessary to use materials that contain a combination of hydroxyapatite with polymers or bioceramics, which significantly improves their osteoinductive properties and promotes cell proliferation [9].

This article describes the diagnostic and therapeutic treatment associated with the occurrence of ectopic teeth in nine dogs. Four cases of teeth located in atypical locations that were additionally complicated by the formation of a dentigerous cyst with severe destruction of the surrounding bone are described in more detail. Special attention was paid not only to the technique of tooth extraction itself but also to the method of protection and healing of the large bone defect after the extraction using hydroxyapatite polymer (HAp) with curdlan.

## 2. Materials and Methods

The group of dogs with ectopic teeth consisted of 9 dogs, which were dogs of the DentalVet practice in Szczecin (Poland). From the point of view of surgical management, in the aforementioned cases the ectopic teeth could be divided into uncomplicated cases and complicated cases. Uncomplicated examples of ectopic teeth included those clinical cases in which the ectopic tooth had been encapsulated in the surrounding connective tissue capsule, without lysis of the bone around the tooth. The complicated cases were more complex cases where, in addition to the ectopic tooth, there was pressure on the surrounding tissues leading to the formation of an alveolar cyst, resulting in lysis of the bone around the cyst. All cases were referred by clinicians as dental dogs with non-specific general symptoms (Table 1).

Bone substitute material

A hydroxyapatite polymer preparation with third-generation curdlan was used to fill all post-extraction cavities. The FlexiOss^®^ Vet preparation used in this study is a material commonly available on the market as a bone substitute material for application in veterinary medicine and has a proven record of biocompatibility. The innovation of this preparation lies in its use of the sugar polymer curdlan, thanks to which it takes on a plastic form after soaking in saline or blood [10]. The plasticity of the material is therefore an advantage, significantly facilitating molding, fitting into the alveolus, and insertion into the cavity through a small bone window. The available literature reports a positive effect of HAp preparations with curdlan on the healing of post-extraction alveoli in dogs, which is why this polymer was chosen to fill cavities after ectopic tooth extraction [11].

Preparation of the bone substitute material

Hydroxyapatite polymer with curdlan (FlexiOss^®^Vet, Medical Inventi, Lublin, Poland) was removed, using sterile procedures, from the sterile envelope, and the shape was placed in a vessel containing a saline solution that completely covered the biomaterial shape. The time taken for the material to soak and become malleable depended on its size and was indicated in the product information leaflet. After waiting the recommended amount of time and confirming the plasticity of the material, molds reflecting the bone defect were cut with sterile scissors and tweezers and implanted in the post-extraction defects. In each case, clinical records and radiological assessment before and after the procedure were reviewed at the end of treatment. After surgery, the dogs received amoxicillin with clavulanic acid (Synulox, Zoetis) at a dose of 15 mg/kg 2× a day for 7 days, buprenorphine (Bupredine Multidose, Dechra) at a dose of 10–20 mcg/kg for the first 3 days, and enflixib (Daxocox, Virbac) at 8 mg/kg (single administration, effective for 7 days) as an analgesic. All procedures were carried out in accordance with the law and international recommendations for best practice in veterinary clinical care in all cases and in accordance with Polish regulations (Art. 1(2)(1) Journal of Laws 2015 item 266). The consent of the local ethics committee was not required. Owners consented to the inclusion of information about their animals in this study.

### 2.1. Surgical Management of Uncomplicated Cases

The dogs qualified for surgery on the basis of clinical examination and oral X-ray. The method of anesthesia, due to the diversity of the dogs, was selected on the basis of an individual protocol, which took into account each dog’s age and general condition, as well as the location of the ectopic tooth. The CLP, using a piezotome (Piezotome^®^ Cube by Acteon), was adopted as the surgical technique in order to minimize as much as possible the tissue loss necessary to achieve surgical access to the tooth. After dissecting the surgical flap within the mucosa, creating access in the bone, and removing the ectopic tooth, the connective tissue cyst in which it was located was carefully lanced. Surrounding bone fragments were taken for histopathological examination to see if any cyst cells remained in the bone around the tooth, which could cause complications in the form of impaired healing and fibrous tissue proliferation. The bone defects were subsequently filled with the widely available FlexiOss^®^Vet (hydroxyapatite polymer material with curdlan). This material has bone-forming properties and, thanks to the use of curdlan, becomes malleable when soaked in saline, making it unnecessary to enlarge the working hole in the bone to implant the bone substitute material. The mucosal flap was then sutured with a single suture using 4-0 monofilament absorbable material. Radiological follow-up was carried out 28 days after the procedure.

### 2.2. Management of Complicated Cases

Case series
Case 1

**Figure 2:**
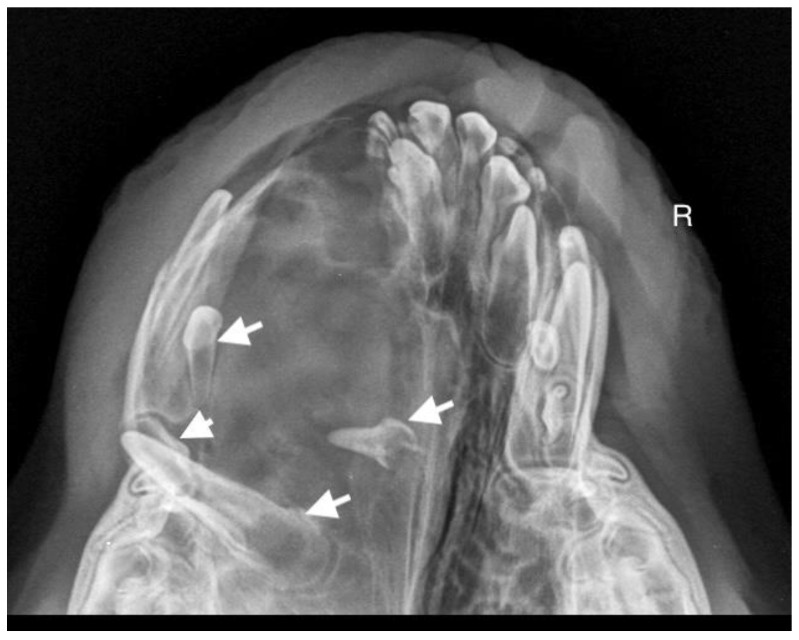
X-ray in dorsal–ventral projection before surgery. On the left side, four ectopic teeth (marked with arrows) with the formation of a dentigerous cyst are visible.

**Figure 3:**
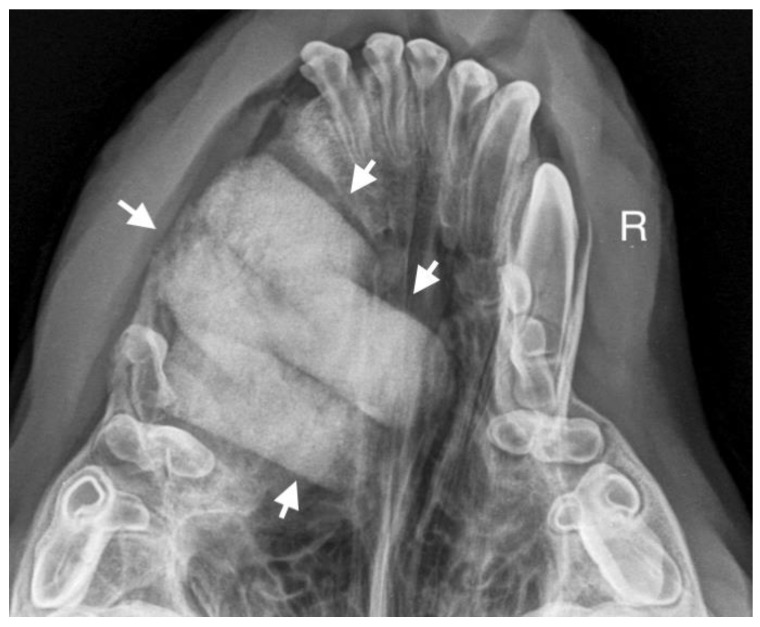
Condition after extraction of ectopic teeth. On the left side, a scaffold for the bone formed of hydroxyapatite polymer material is visible (marked with arrows).

**Figure 4:**
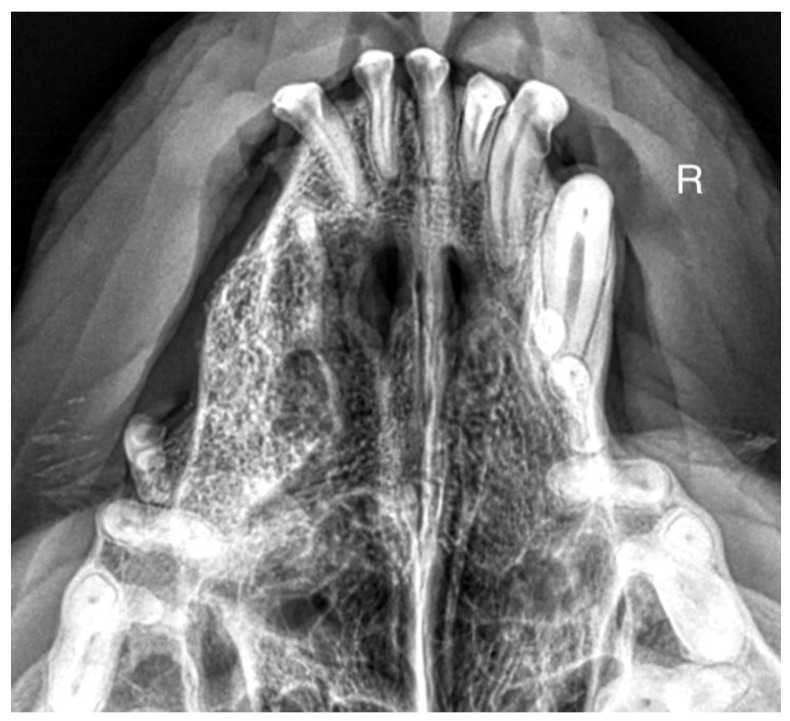
Condition 12 months after the surgery to remove ectopic teeth. On the left side, a properly formed jawbone and nasal cavity are shown.

A 7-month-old male Rottweiler was referred for consultation because of severe swelling on the left side of the jaw, with a suspected jaw tumor. Clinical examination revealed no abnormalities in the aforementioned lymph glands and no soreness on palpation. Intraoral examination on the left side revealed severe asymmetry, with enlargement of the facial soft and hard tissues, an absence of permanent teeth, and buccal displacement of deciduous teeth. On the left side, rhinoscopic examination revealed normal anterior nostrils and a left nasal aperture with a lack of patency from the level of the nasal bony inlet to the posterior nostrils. A roentgenogram in the dorsal–ventral projection showed the presence of four ectopic teeth in the maxilla, with the formation of a dentigerous cyst (Figure 2). Using the CLP technique, the ectopic teeth were removed and the cyst walls were lanced. The bone loss caused by the cyst was 7.3 cm × 4.6 cm, and the jawbone left behind was too thin to provide a scaffold for the surrounding tissues and to ensure nasal cavity patency. From a 5 cm pellet of hydroxyapatite polymer material with curdlan previously soaked in saline (according to the manufacturer’s instructions), longitudinal flaps of 5 cm long, 1 cm wide, and 0.1–0.2 cm thick were cut and placed in the bony defect to strengthen the bony scaffolding of the jaw and nasal septum (Figure 3). The gingival flap was sutured with a single suture using 4-0 monofilament material. The first radiological follow-up was performed after 4 weeks, but due to the size of the defect and the size of the pellets of material used, the hydroxyapatite obscured the structures in the nasal cavity, preventing accurate assessment. A rhinoscopic examination revealed patency of the left nasal cavity. It was decided to conduct a radiological follow-up 6 months after the procedure, but the owner, for personal reasons, did not attend the follow-up appointment. After 12 months, another roentgenogram was performed, where a properly healed jawbone with closure of the cyst cavity could be observed. The nasal septum was formed properly, and the resulting bony scaffolding resulted in patency of the left nasal aperture, along with normal airflow on the left side (Figure 4).

Case 2

**Figure 5:**
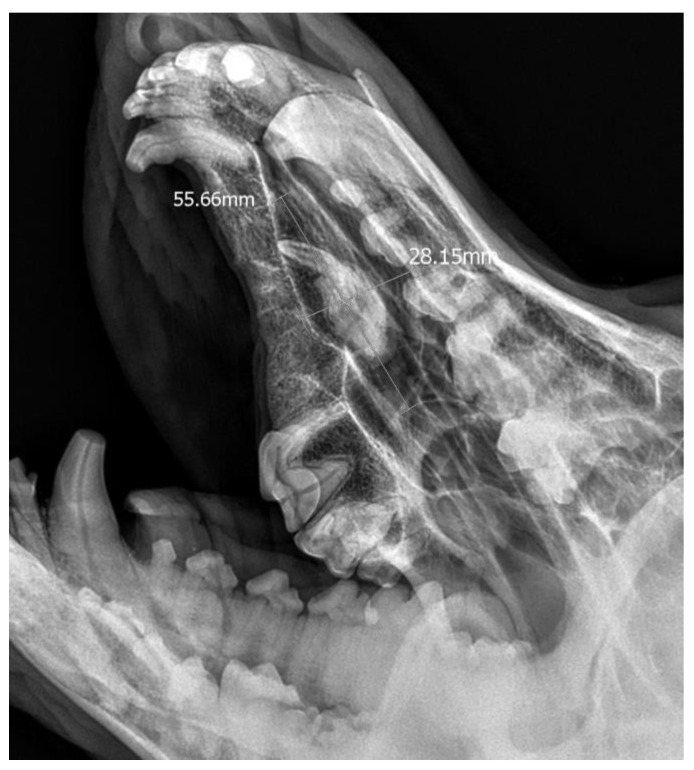
X-ray in oblique head projection. The image shows an ectopic tooth along with an alveolar cyst (the dimensions of the cyst are marked).

**Figure 6:**
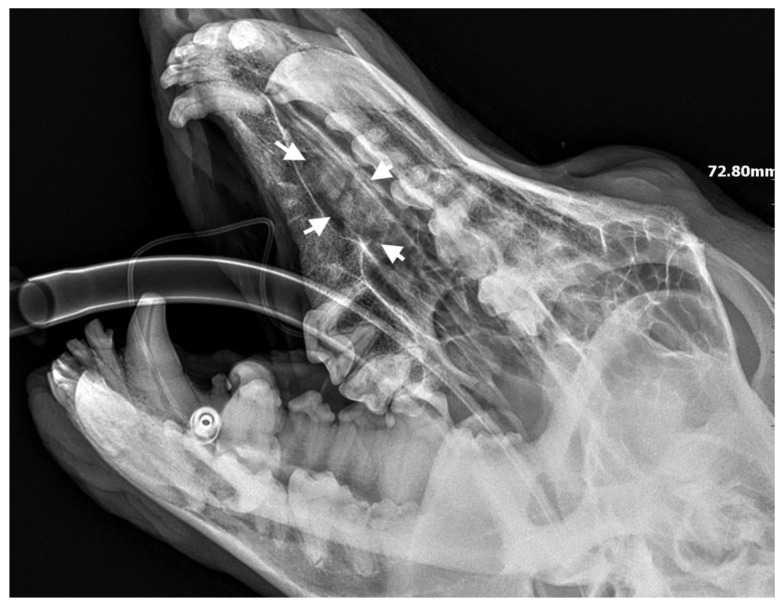
X-ray showing the condition of the jawbone 28 days after tooth extraction. The bone defect is filled with bone tissue without signs of inflammation (marked with arrows).

A 3-year-old German Shepherd dog was referred with a suspected periapical abscess of a tooth on the right side. The medical history of the pet client reported that the dog had been treated for chronic rhinitis for 6 months. On clinical examination, tooth 104 (right maxillary fang) was observed to be missing, with no wounds or fistulas in the oral cavity. Rhinoscopic examination showed features of chronic rhinosinusitis, with convexity of the nasal wall mucosa on the right side of the nasal cavity, without proliferative changes or foreign bodies. Radiological examination revealed the presence of an ectopic tooth located outside the alveolar process in the region of the maxillary cranium. Its shape was not characteristic of any normal tooth. It had a soppy crown and an incompletely formed root. The tooth was surrounded by a dentigerous cyst measuring 5.6 × 2.8 cm (Figure 5). After tooth extraction using the CLP technique and cyst lysing, the bone defect was filled with a hydroxyapatite polymer material previously soaked in 0.9% NaCl. The plasticity of the material was exploited so that it was possible to fill the bone defect with the material through a dissected bone window of 3 cm in diameter without enlarging the bone defect. A follow-up X-ray 28 days after the procedure showed normal filling of the defect with bone, without signs of inflammation or granulation formation within the jawbone. On clinical examination, resolution of nasal discharge problems and the absence of features of inflammation within the nasal cavity were observed (Figure 6).

Case 3

**Figure 7:**
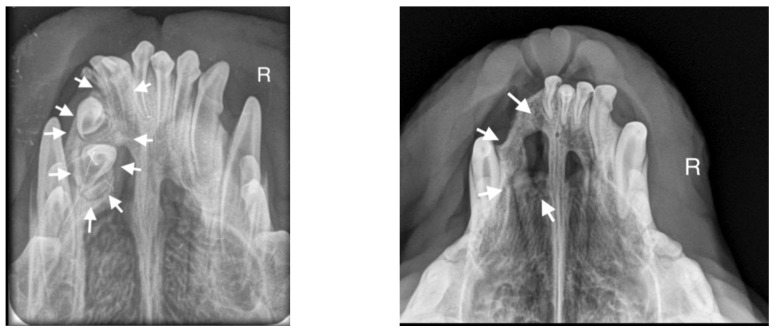
On the left side, the teeth with abnormal eruption are marked with arrows on the X-ray. The right radiograph shows the status 28 days after surgery. Properly formed bone tissue can be seen, with no signs of inflammation or alveolar bone atrophy (marked with arrows).

**Figure 8:**
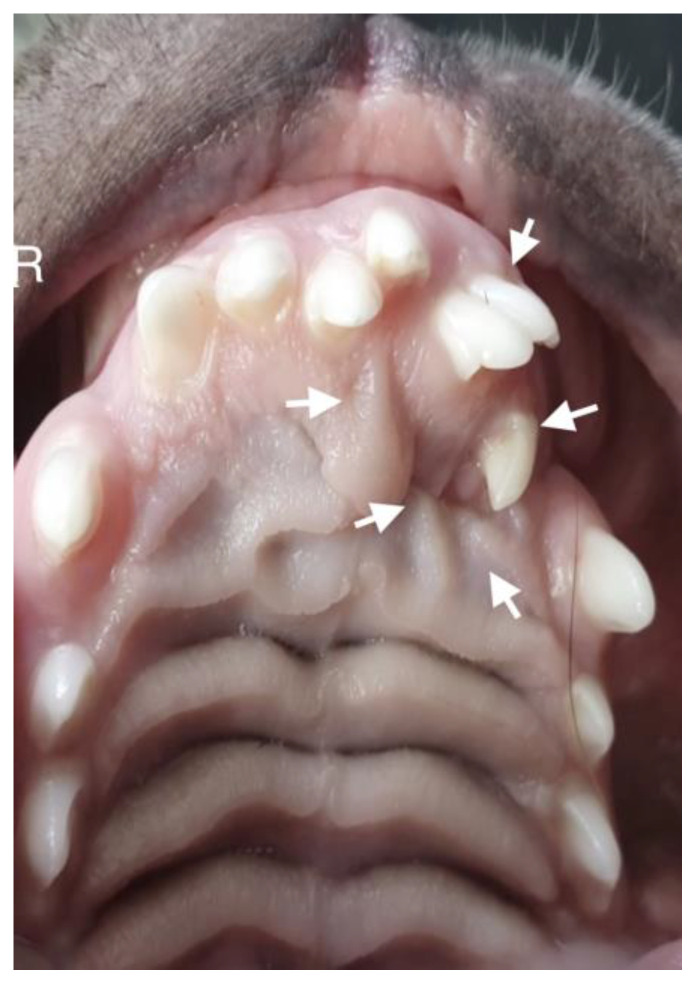
Intraoral photograph of the dog. On the left side of the dog’s mouth, chaotic tooth growth (marked with arrows) and atypical curvature within the palatal crease with displacement to the left side of the incisor papilla are visible.

A 10-month-old American Staffordshire Terrier dog was referred for an orthodontic consultation due to abnormal alignment of the maxillary teeth and the resulting bite injuries. Clinical examination revealed three properly aligned incisors on the right side and four incisors (including one tooth with a double crown) on the left side arranged chaotically (Figure 7). Intraoral examination revealed an abnormal palatal crease in the projection of teeth 103–203 and a hard and painful protrusion about 3 cm in diameter on the left side. The roentgenogram showed the presence of three additional incisors, including two unerupted ones, growing in the opposite direction to the alveolar process. The double tooth on the X-ray appeared to be a fused tooth (tooth fusion occurs as a result of physical force or pressure on an adjacent tooth’s bud, leading to the contact of the two tooth buds and their fusion before calcification) [12]. The abnormal growth of the incisors on the left side caused pressure on the incisal bone, along with skewing of the nasal septum to the right side (Figure 8). The third incisor, along with an additional third incisor, showed radiographic features of a dentigerous cyst. A decision was made to perform open extraction of the ectopic teeth using the CLP method.

The bone cavity, after such a large open extraction, was 3.0 cm × 4.2 cm; so, the decision was made to implant polymeric hydroxyapatite with curdlan soaked in 0.9% NaCl into the areas of bone loss. The flap was sutured with 4-0 monofilament single sutures. A follow-up radiograph was taken 28 days after surgery. On clinical examination, the soft tissues were healed properly. On the follow-up radiograph, the bone tissue was healed properly, with new bone formation.

Case 4

**Figure 9:**
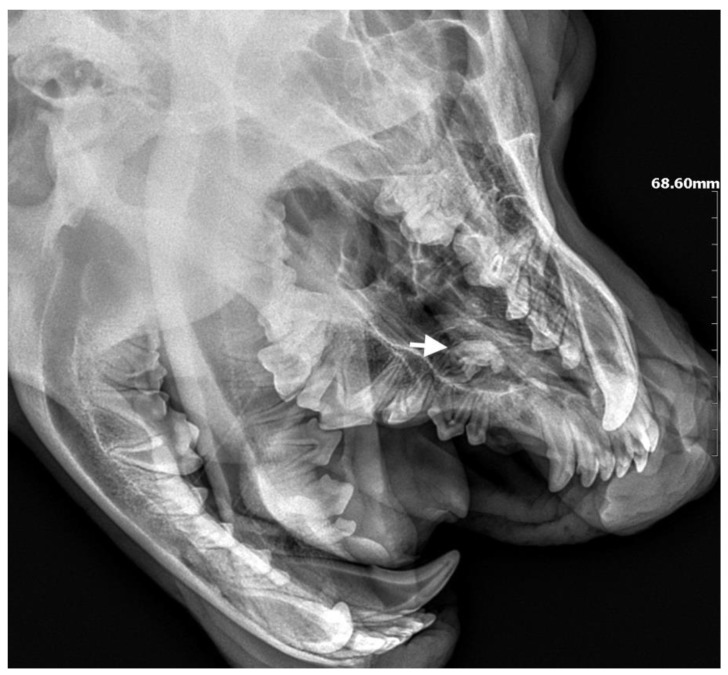
Preoperative radiograph. At the level of teeth 205–207, a shadow with saturation characteristic of tooth tissue confirmed the presence of an ectopic tooth (marked with an arrow).

**Figure 10:**
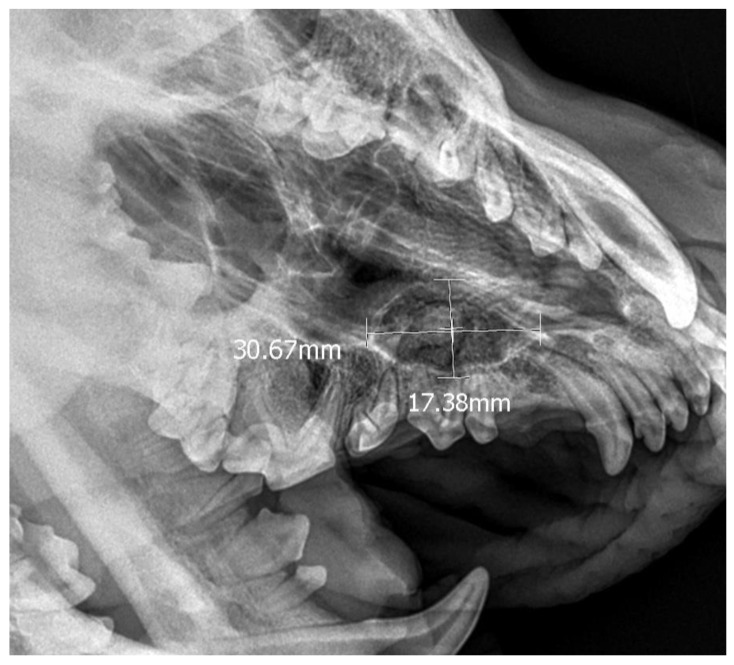
X-ray confirming the presence of an ectopic tooth along with a 3 cm × 1.7 cm dentigerous cyst on the left side.

**Figure 11:**
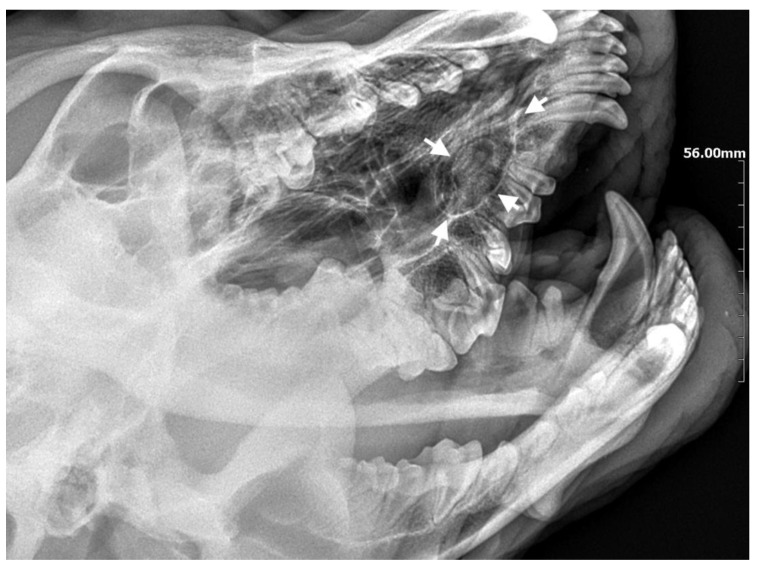
Follow-up roentgenogram showing normal bone recovery at the post-extraction defect site 28 days after the procedure (marked with arrows).

A 14-month-old female Cane Corso was referred for dental diagnosis due to the absence of tooth 204 and periodic swelling of the left suborbital region. The dog had been treated ophthalmologically for 4 months for recurrent conjunctivitis of the left eye and increased epiphora. Ophthalmologic examination revealed obstruction of the left nasolacrimal duct, the cause of which was attributed to recurrent inflammation of the infraorbital region. The roentgenogram in the oblique projection, in the view of teeth 205–207, showed the presence of a shadow with saturation characteristic of tooth tissue, with an atypical shape (Figure 9). The roentgenogram confirmed the presence of an ectopic tooth, along with a dentigerous cyst measuring 3 cm × 1.7 cm in the area of the maxillary cranium and the fossa of the lacrimal sac (Figure 10). The tooth was accessed using the CLP technique, then an opening was drilled into the anterior wall of the maxillary cusp and the horizontally lying ectopic tooth 204 was visualized. After removal of the tooth, the lumen of the bone defect was reviewed and the empty space was filled with hydroxyapatite polymer material with curdlan. The bone substitute material was soaked in 0.9% NaCl before surgery, making it malleable and allowing the bone defect to be filled through a 2 cm bone window. The mucosa was sutured with a 4-0 absorbable monofilament suture. Radiological follow-up was carried out 28 days later, which showed that the defect was filled with newly formed bone. No inflammatory changes or granulation tissue formation were found in the area of the defect (Figure 11).

## 3. Results

The presence of ectopic teeth was found in nine pet clients, representing 0.18% of the total number of clients of the practice. Among the nine dogs, there were five males and four females of different breeds, aged between 7 months and 3 years, with a weight range of 8 to 40 kg. In eight of the dogs, an ectopic tooth was diagnosed in the jaw, while in one dog it was present in the mandible. Three of these dogs were brachycephalic breeds (Cane Corso, Rottweiler, Amstaff), while the remaining dogs were mesocephalic breeds (two German Shepherds, Airedale Terrier, Dachshund, Miniature Schnauzer, hybrid). The cases of ectopic teeth were divided into uncomplicated ectopic teeth (five dogs, 55.56% of clients) and complicated ectopic teeth (four dogs, 44.44% of dogs). In five dogs, the occurrence of ectopic teeth did not result in the destruction of the surrounding bone, and they were therefore classified by the authors as uncomplicated tooth cases. In three cases, there was a dentigerous cyst that had had a highly destructive effect on the surrounding bone, causing significant defects within the surrounding tissues. In one case, the ectopy was due to the presence of extra teeth (Amstaff dog), which also caused cyst formation.

The pet clients in the uncomplicated ectopic teeth group underwent standard extraction of these teeth. The dogs in the group of ectopic teeth with complications underwent the CLP technique for tooth extraction, and the large bone defect created after curettage of the connective tissue capsule was protected with the hydroxyapatite polymer material FlexiOss^®^Vet. The CLP technique performed well as a method of ectopic tooth extraction in these dogs, and healing was without complications. However, this does not rule out possible complications. The study should be continued on a larger number of animals to determine the potential postoperative complications and the manners of preventing them.

X-ray examinations performed 28 days after surgery (and in one case, after 12 months) showed the bone cavities were filled with tissue with the same X-ray permeability as bone, suggesting the formation of bone tissue to fill the cavity. The study described herein was non-experimental, so there was no indication for histopathological sampling of dogs and bone regeneration was assessed by X-ray. Determining the degree of bone regeneration that can be achieved using hydroxyapatite polymer with curdlan requires additional research.

## 4. Discussion

Ectopic teeth are a rare anomaly faced by veterinary surgeons and dentists. Ectopic teeth can occur anywhere in the craniofacial region, as well as in other parts of the body, and the pressure they exert on tissues causes non-specific general signs of their location. They are more commonly diagnosed in small-breed dogs, suggesting that an abnormal relationship between the size of the teeth and the bone in which they are located may be a factor in causing ectropion [13,14]. This disorder in animals affects permanent teeth, with the most common occurrence being in the maxilla (69.57%) and it occurring less commonly in the mandible (30.43%). The most common teeth erupted ectopically are incisors (40.62%), followed by canines (18.75%). The most common ectopic teeth in the mandible are the first premolar (42.86%) and the third molar (42.86%) [14]. In the authors’ own study, this disorder occurred in the maxilla in 88.88% of the cases and in the mandible in 11.12%. The tooth that was most frequently diagnosed as an ectopic tooth was the fang (77.77%), followed by the incisor (22.22%), and, least frequently, the premolar (11.11%) and molar (11.11%). More than one ectopic tooth was found in 22.22% of the pet clients. Klim et al. performed a detailed analysis of the abnormalities associated with ectopic teeth in dogs [14]. In this study, the authors reported 35 cases of ectopic teeth, combining the problems of both unerupted teeth located in atypical sites for teeth and those whose eruption problems occurred due to orthodontic reasons. The combination of these cases led to the higher number of reports. In the described cases, we applied stricter criteria for the classification of an ectopic tooth; therefore, the number of dogs with this condition was smaller. We rejected cases of orthodontic abnormalities and focused on teeth occurring in unusual locations.

The problem with accurately classifying ectopic teeth lies in the definition of an ectopic tooth. According to Bodenmark et al., the term refers to teeth that erupt in areas where teeth do not normally occur [12]. The qualification of cases for the present article was based on the definition from Bodenmark due to its frequent use in describing similar cases in human dentistry. Ectopic eruption, on the other hand, according to Colyer et al., refers to teeth that are positioned abnormally, which may therefore include not only teeth that occur in unusual locations but also teeth with abnormal orthodontic alignment [15]. This definition was used by Klim et al., cited above.

An abnormal tooth count should prompt the clinician to perform a follow-up X-ray to determine the location of the tooth or the absence of the tooth. However, these procedures are not routinely performed in veterinary practices, especially when the carer reports symptoms uncharacteristic of dental problems. Pet guardians, unless the dog is a breeding or working animal, rarely pay attention to the correct number of teeth in the mouth. The dogs involved in the study were referred by their handler for extraction of ectopic teeth that were diagnosed incidentally in a diagnostic process involving other conditions. The authors recognize that due to the rarity of ectopic teeth and the similarity of clinical signs to various diseases, these cases can pose a major diagnostic and therapeutic challenge for veterinary clinicians [16]. Voelter-Ratson et al. described the case of a dog with nasal duct obstruction with the presence of an ectopic tooth [17]. This case perfectly describes the diagnostic complexities that a clinician faces before a proper diagnosis can be made. Severe epiphora and recurrent conjunctivitis were not among the pathognomonic symptoms of an ectopic tooth, so the first diagnostic steps were directed towards the diagnosis of an ocular disease and the dog was first examined ophthalmologically. Similar diagnostic steps could be found in the case of one of the patients from human medicine described in this article, where an ectopic tooth was located in the region of the maxillary cingulum and the fossa of the lacrimal sac (case 4). In their clinical case report, Alfayez et al. gave the example of a patient with difficulty breathing and recurrent airway inflammation, often complicated by bacteria. An ENT examination of the patient showed a curvature of the nasal septum and a transparent mass on the floor of the nasal cavity. It was not until a CT scan was carried out in preparation for the patient’s rhinoplasty procedure that the true cause of the patient’s ENT problems, the presence of an ectopic tooth, was revealed [18]. The description of the clinical symptoms, even though this case was in human medicine, was very similar to case no. 2 described in this article, where an ectopic tooth was located in the maxillary cusp. In contrast, Lambade et al. presented the case of a patient where an ectopically located mandibular tooth led to an extraoral fistula, a scar below the earlobe and, following osteomyelitis of the mandibular condyle, to vestibular symptoms. The patient was primarily diagnosed otologically and neurologically. A radiological examination of the head, where an ectopic tooth was visualized, was used to make a definitive diagnosis [19].

Although an ectopic tooth is most often asymptomatic, without surgical removal it can develop into a cyst or tumor. Enlargement of the cyst is accompanied by resorption of the adjacent bone, which can lead to severe weakening of the jaw or mandibular bone. Therefore, ectopic teeth should be removed by open extraction and the entire epithelial lining of the cyst carefully removed to avoid recurrence and complications. Reports have also been published stating that the lining may undergo neoplastic metaplasia and, although these data are limited, it is always advisable to carry out histopathological examination [20].

The differential diagnosis, especially in the case of ectopic teeth located in an alveolar cyst, should include alveolar keratocysts and alveolar neoplasms (multiple myeloma, Pindborg tumor, dentoma, alveolar fibroma, and cementoma) and non-neoplastic lesions, such as retention cysts and mucoceles [21,22]. The lack of a specific definition of an ectopic tooth has led authors to consider the orthodontic treatment options, in addition to surgical treatment approaches, for ectopic teeth [15]. In human dentistry, the Caldwell–Luc Procedure (CLP) is used when removing ectopic teeth from hard-to-reach areas, as it gives good access to the tooth. The disadvantage of this technique is the creation of a large bone defect, and thus an increased risk of complications during and after the procedure [23]. In human medicine, ENT surgeons and otologists are increasingly turning to a less invasive technique using an endoscope, but the cost of endoscopes and the lack of training facilities in this field prevent the routine use of endoscopy [24]. The choice of surgical access generally depends on the experience and preference of the surgeons. The use of the CLP technique in canine dentistry requires the operator to be particularly careful due to the proximity of the infraorbital foramen located above the second (or, depending on the skull structure, third) maxillary premolar tooth and the possibility of damaging the blood vessels and nerves located there. For this reason, the procedure carries a higher risk in comparison to human dentistry. In human dentistry, special attention is paid to the use of intraoral access, which allows facial nerve damage to be avoided, as pointed out by Pace et al. and Calliet et al., with whom the authors of this article agree [25,26].

Postoperative follow-up is necessary until the defect is filled with bone tissue. Early post-operative complications include severe bleeding, impaired healing of the bone, post-extraction alveolus, and even the development of necrotizing osteitis, or dry socket, although this name is not particularly accurate in the case of the location of the ectopic tooth outside the alveolar bone. The more common complications are late complications, which can occur around two weeks after surgery and cause problems for the pet client for the rest of his or her life. The weakened bone area can cause pathological fractures within the weakened tissue. In the case of an ectopic tooth occurring in the mandible, this means pathological fractures of the mandible, and in the case of the maxillary bone, complications can lead to difficulties breathing, prevention of the free flow of tears through the nasolacrimal duct, and, in extreme cases, orbital remodeling and associated ophthalmological problems. Large bone defects that are not filled with bone-replacement material after surgery will be replaced with highly collagenized connective tissue that will overgrow in the form of cauliflower-shaped formations in the oral cavity, making it difficult to take in food and becoming a site for mechanical damage, bleeding, and inflammation.

Although the authors do not address this issue in their reports, securing the defect with bone substitute material and, if possible, accelerating healing appears to be essential in veterinary clients, who will not be affected in the same way by postoperative wound complications [17,18,19,20]. With multiple or deep alveolar bone defects and in dogs with a thin periodontal biotype, not only dimensional changes in bone but also soft tissue recession may occur [27]. In order to prevent such complications, HAp-based mineral preparations are increasingly used as a scaffold for newly formed bone at the defect site. Studies by Misztal-Kunecka et al. using a hydroxyapatite polymer preparation with curdlan showed a significant acceleration of bone regeneration from 56 to 28 days after surgery; hence, the use of the abovementioned material seems to be essential in order to reduce the number of postoperative complications [11]. In the treatment of the dogs described in this article, a modern, third-generation HAp implant preparation was used (hydroxyapatite polymer with curdlan) [28]. This is the only preparation available on the market that takes a plastic form after soaking, which is particularly applicable in cases where the ectopic tooth has been successfully removed to create a small bone window, without the need for invasive intervention into the bone surrounding the ectopic tooth. Commercially available pellet, powder, and granule formulations are difficult to use to effectively fill the post-extraction alveolus tightly. After curettage of the alveolus, especially in cases complicated by the formation of an alveolar cyst, severe bleeding can occur due to bone irritation from the bone spoon. For this reason, implantation of a bone substitute material in powder form is often very difficult or impossible. When implanting material in the form of pellets and granules, it is difficult to achieve a seal between the individual structures, which can result in voids. On the other hand, failure to fill the defect tightly with the bone substitute material may be associated with the formation of granulation tissue and impaired healing of the post-extraction wound [29,30].

## 5. Conclusions

The polymeric hydroxyapatite with curdlan used in the described procedures performed well when filling the large bone cavities that arose during the extraction of ectopic teeth in dogs. Due to the plastic properties of hydroxyapatite polymer with curdlan, it was possible to easily, tightly, and permanently implant the material without enlarging the resulting bone defect. Follow-up radiographs at 28 days after material implantation (in the case of eight dogs) and at 12 months (in the case of the dog with the largest bone defect) showed radiological features of bone regeneration. Clinical examination at both the early and late stages showed no postoperative complications.

## Figures and Tables

**Table 1:** Symptoms and associated site of ectopic teeth.

	Dog 1	Dog 2	Dog 3	Dog 4	Dog 5	Dog 6	Dog 7	Dog 8	Dog 9
**Age**	10 months	4 years	1 years	2 years	1.5 years	7 months	14 months	10 months	3 years
**Sex**	female	male	female	male	male	male	female	female	male
**Breed**	Dachshund	Hybrid	Miniature Schnauzer	German Shepherd	Airedale Terrier	Rottweiler	Cane Corso	American Staffordshire Terrier	German Shepherd
**Systemic pathologies**	no	no	no	no	no	no	no	no	no
**Location of** **an ectopic tooth**	jawbone in the alveolar bone of the region of tooth 209	jawbone in the alveolar bone of the area of tooth 204	mandibular bone of the area of tooth 404	jawbone in the alveolar bone of the area of tooth 104	jawbone in the alveolar bone of the area of tooth 204	from the height of the nasal bony inlet to the posterior nostrils	the area of the maxillary cranium and the fossa of the lacrimal sac	maxillary incisal bone	maxillary craniofacial region
**Number of ectopic teeth**	1	1	1	1	1	4	1	3	1
**Occurrence of dentigerous cysts**	No	No	No	No	No	Yes	Yes	Yes	Yes
**Clinical symptoms**	No additional symptoms, unerupted tooth 209	No additional symptoms, unerupted tooth 204	No additional symptoms, unerupted tooth 404	No additional symptoms, unerupted tooth 104	No additional symptoms, unerupted tooth 204	Severe asymmetry with enlargement of the soft and hard tissues of the face	Swelling of the left suborbital region, recurrent conjunctivitis of the left eye, and increased epiphora	Abnormal position of palatal creases, hard and painful flexion on the left side of the jaw	Missing tooth 104, chronic nasal mucositis on the right side

## Data Availability

Dataset available on request from the authors—the raw data supporting the conclusions of this article will made available by authors on request.
